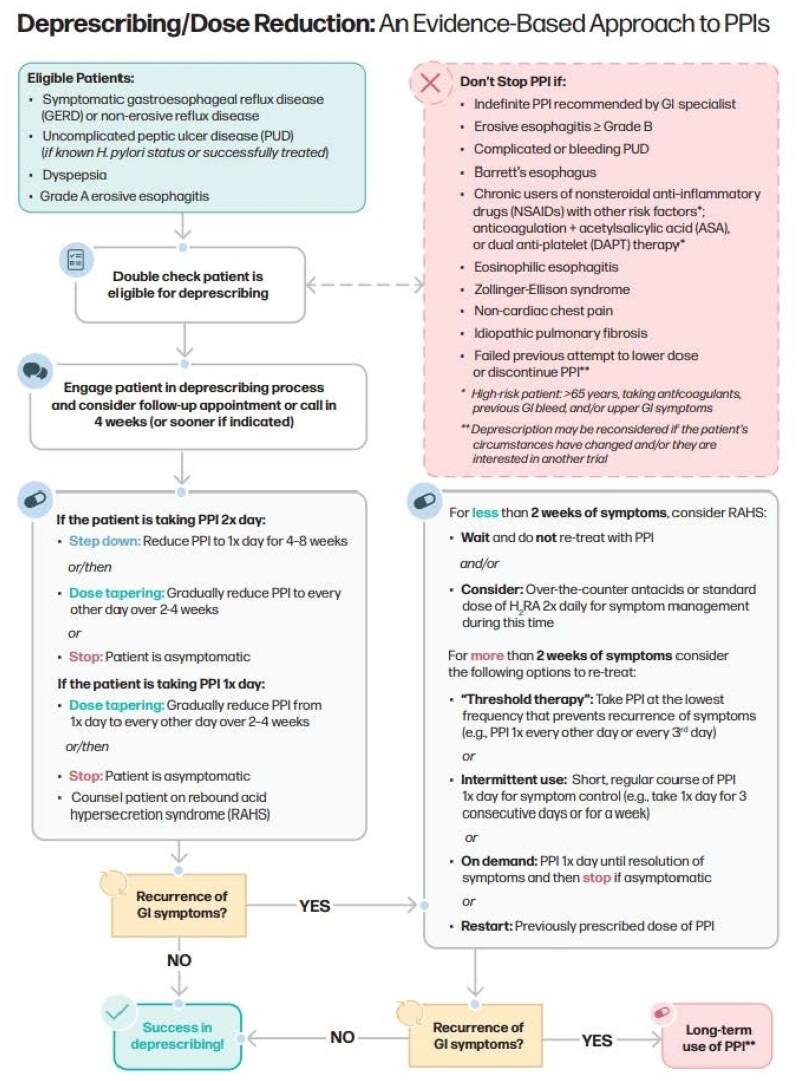# Poster Session I - A107 CHOOSING WISELY: THE NEW AND IMPROVED PPI TOOLKIT: ASK WHY FOR PPIS

**DOI:** 10.1093/jcag/gwaf042.107

**Published:** 2026-02-13

**Authors:** S Veldhuyzen Van Zanten, E v Bland, M Magaz, O Ostrow

**Affiliations:** Medicine, University of Alberta, Edmonton, NS, Canada; University of Calgary Cumming School of Medicine, Calgary, AB, Canada; University of Toronto, Toronto, ON, Canada; University of Toronto, Toronto, ON, Canada

## Abstract

**Background:**

Choosing Wisely Canada (CWC) is the national organization whose aim is to reduce unnecessary tests and treatments in Canada. The supporting *CWC toolkit “Bye, Bye PPIs”* was launched in 2017. It has been one of the most frequently downloaded toolkits. However, this toolkit was outdated and missed appropriate prescribing information on PPI use, including conditions such as (symptomatic) Gastro Esophageal Reflux Disease (GERD) and dyspepsia, and lacked an evidence based approach for deprescribing/dose reduction.

**Aims:**

To develop an up to date evidence based toolkit to support prescribers and pharmacists in implementing effective interventions to optimize PPI prescribing for adults and adolescents.*1)* Prescribing PPIs only for appropriate indications, durations and doses, *2)* Deprescribing PPIs when there is no indication for long-term use, and *3)* Engaging patients in reducing unnecessary continuous PPI use when there is no ongoing indication, the dose can be lowered, or no clear benefit is evident.

**Methods:**

A core group of four individuals completed a literature review, Fishbone Diagram and Driver Diagram to better understand the existing gaps, current barriers to appropriate prescribing and describing, and effective tools to address these. Widespread input was obtained from interest holders including Family Physicians, Gastroenterologists, Internal Medicine Physicians, Pharmacologists and Quality Improvement Specialists. Consensus on final recommendations and their supporting tools were reached over a 6-month period.

**Results:**

The updated toolkit (https://choosingwiselycanada.org/toolkit/ask-why-ppis/) includes a table with appropriate prescribing and duration for symptom-based treatment (e.g. dyspepsia), endoscopy-based indications (e.g. peptic ulcers), prophylaxis (e.g. NSAIDS), and rare conditions (e.g. ZE-syndrome) and whether the patient is a candidate for deprescribing. The table includes recommendations on starting dose, need for long-term use, whether step up to bid dosing can occur. A Flowchart is also depicted describing the safe process for dose reduction or deprescribing in eligible patients. See Fig 1. The toolkit includes a “myth busters” section to guide decision-making process of healthcare providers (e.g., “PPIs twice a day controls reflux better than once a day”) and a patient information handout for PPI information and symptom management.

**Conclusions:**

PPIs are among the most widely used medications worldwide. While generally safe and effective, PPIs are often prescribed for a longer duration or at a higher dose than guidelines recommend. The updated CWC toolkit “ASK WHY FOR PPIs” is an evidence based guide that can easily be utilized by busy clinicians highlighting explicit recommendations on indication, dosing, and safe deprescribing of PPIs, and for patients to reduce low-value care.

Deprescribing/Dose reduction: an evidence based approach to PPIs

**Funding Agencies:**

NoneNA